# Short-term effects of deoxynivalenol, T-2 toxin, fumonisin B1 or ochratoxin on lipid peroxidation and glutathione redox system and its regulatory genes in common carp (*Cyprinus carpio* L.) liver

**DOI:** 10.1007/s10695-020-00845-1

**Published:** 2020-07-02

**Authors:** Benjámin Kövesi, Szabina Kulcsár, Erika Zándoki, Judit Szabó-Fodor, Miklós Mézes, Krisztián Balogh, Zsolt Ancsin, Csilla Pelyhe

**Affiliations:** 1 Department of Nutrition, Szent István University, Gödöllő, H-2103 Hungary; 2 Hungarian Academy of Sciences-Kaposvár University- Szent István University, Mycotoxins in the Food Chain Research Group, Kaposvár, H-7400 Hungary

**Keywords:** Deoxynivalenol, T-2 toxin, Fumonisin B_1_, Ochratoxin A, Common carp, Glutathione redox system

## Abstract

The effects of a single oral dose of 1.82 mg kg^−1^ bw of T-2 and HT-2 toxin (T-2), 1.75 mg kg^−1^ bw deoxynivalenol (DON) and 15-acetyl DON, 1.96 mg kg^−1^ bw fumonisin B_1_ (FB_1_) or 1.85 mg kg^−1^ bw ochratoxin A (OTA) were investigated in common carp juveniles on lipid peroxidation, the parameters of the glutathione redox system including the expression of their encoding genes in a short-term (24 h) experiment. Markers of the initiation phase of lipid peroxidation, conjugated dienes, and trienes, were slightly affected by DON and OTA treatment at 16-h sampling. The termination marker, malondialdehyde, concentration increased only as an effect of FB_1_. Glutathione content and glutathione peroxidase activity showed significantly higher levels in the T-2 and FB_1_ groups at 8 h, and in the DON and FB_1_ groups at 16 h. The expression of glutathione peroxidase genes (*gpx4a, gpx4b*) showed a dual response. Downregulation of *gpxa* was observed at 8 h, as the effect of DON, FB_1_, and OTA, but an upregulation in the T-2 group. At 16 h *gpx4a* upregulated as an effect of DON, T-2, and FB_1,_ and at 24 h in the DON and T-2 groups. Expression of *gpx4b* downregulated at 8 h, except in the T-2 group, and upregulation observed as an effect of T-2 at 24 h. The lack of an increase in the expression of *nrf2,* except as the effect of DON at 8 h, and a decrease in the *keap1* expression suggests that the antioxidant defence system was activated at gene and protein levels through Keap1–Nrf2 independent pathways.

## Introduction

Mycotoxins are secondary metabolites of moulds. *Fusarium* moulds produce trichothecene mycotoxins, such as T-2 toxin and deoxynivalenol (DON), and fumonisin B_1_ (FB_1_), while ochratoxin A (OTA) is produced by *Aspergillus* and *Penicillium* species (Placinta et al. 1999; Jørgensen 2005). These mycotoxins contaminate the food and feed products and by-products, and their oral intake has adverse effects on human and animal health, including fish (Anater et al. 2016). Nevertheless, the effects of these globally significant mycotoxins are not fully described in fish species, and the results show a great variety in different fish species. According to a recent worldwide survey, DON was found to be the most prevalent mycotoxin in cereal grains, which occurs at high levels in continental areas (Biomin 2020).

T-2 toxin is considered as one of the most toxic trichothecene (Bamburg et al. 1968). The chemical structure of both T-2 toxin and DON contains an epoxy group, which makes them reactive (Krska et al. 2001). T-2 toxin and DON inhibit protein, DNA, and RNA synthesis (Holladay et al. 1995), which can affect the immune system (Kidd et al. 1995). Additionally, DON has adverse effects on feeding behaviour through the serotoninergic system as a neurotoxic compound (Fioramonti et al. 1993) and may also be related to the effects on cell signalling processes (Leathwood 1987). FB_1_ toxicity is linked to the inhibition of the ceramide biosynthesis and more complex sphingolipids through the inhibition of ceramide synthase (Meredith et al. 1998; Goel et al. 1994). OTA is nephrotoxic, hepatotoxic, neurotoxic, teratogenic and immune-toxic in vivo and in vitro model systems (Heussner and Bingle 2015). OTA inhibits the phenylalanine tRNA synthesis (Konrad and Röschenthaler 1977) and causes DNA damage (Lioi et al. 2004). Some toxic effects of T-2 toxin, DON, FB_1_ and OTA in fishes are summarised in Table 1.

**Table 1:** Toxic effects of T-2 toxin, DON, fumonisin B1 and ochratoxin A in fishes

Mycotoxin	Fish species	Effects	References
T-2 toxin	*Oncorhynchus mykiss* *Ictalurus punctatus* *Cyprinus carpio*	Retarded growth Haematocrit ↓ Haemoglobin ↓ Retarded growth Mortality ↑ Retarded growth Mortality ↑ Immune response ↓	Poston 1983 Manning et al. 2003a Balogh et al. 2009 Modra et al. 2020
DON	*Oncorhynchus mykiss* *Cyprinus carpio*	Feed refusal Retarded growth Feed efficiency ↓ Immune response ↓ Liver damage	Woodward et al. 1983 Hooft et al. 2011 Pietsch et al. 2015 Matejova et al. 2015 Pietsch et al. 2014
Fumonisin B_1_	*Ictalurus punctatus* *Clarias gariepinus* *Oreochromis niloticus* *Oncorhynchus mykiss* *Cyprinus carpio*	Body weight ↓ Disease resistance ↓ Haematocrit ↓ red cell count ↓ WBC count ↓ Antibody production ↓ Inflammation in the liver, kidney, and the intestine Retarded growth Serum protein level ↓ Lesions in the liver, endocrine, and exocrine pancreas, kidney, heart, and brain Weight gain ↓ Haematocrit ↓ Liver sphinganine ↑ Liver damage	Lumlertdacha et al. 1995 Lumlertdacha and Lovell 1995 Brown et al. 1994 Pepeljnjak et al. 2003 Petrinec et al. 2004 Tuan et al. 2003 Carlson et al. 2001 Pepeljnjak et al. 2003
Ochratoxin A	*Ictalurus punctatus* *Oreochromis niloticus*	Mortality ↑ Weight gain ↓ Haematocrit ↓ Disease resistance ↓ Feed efficiency ↓ Weight gain ↓ Feed efficiency ↓	Manning et al. 2003b Manning et al. 2005 Mansour et al. 2015

Trichothecenes, OTA and FB_1_ are described to affect the intensity of lipid peroxidation and the activity of the biological antioxidant defence in different animal species (Mézes et al. 1998; Surai et al. 2002; Ramyaa and Padma 2013; Wang et al. 2016). T-2 toxin induces apoptosis, which may be related to emerging oxidative stress, which can activate mitochondrial apoptotic pathways (Jaradat 2005). Kravchenko et al. (1989) investigated the effect of T-2 toxin on the xenobiotic transformation in common carp, and a moderate increase was found in the activity of glutathione S-transferase (GST). A long-term feeding trial with 0.52 or 2.45 mg T-2 toxin kg^−1^ feed resulted in an elevated level of reduced glutathione (GSH) and glutathione-peroxidase (GPx) activity, while lipid peroxidation, as measured by the concentration of malondialdehyde (MDA), did not change significantly (Balogh et al. 2009). In another 4-week long feeding study with common carp juveniles (Pelyhe et al. 2016a), T-2 toxin and DON did not modify the parameters of lipid peroxidation. However, in the same study, activities of GPx and GST decreased slightly, but significantly, as the effect of T-2 toxin, but not in the case of DON. GSH concentration showed moderate changes as an effect of DON exposure, but T-2 toxin did not affect. Expression of phospholipid hydroperoxide glutathione peroxidase (*gpx4*) genes showed a different response to mycotoxin exposure. T-2 toxin caused early and late downregulation and midterm upregulation in the case of *gpx4a,* but continuous upregulation was found as an effect of DON. Both trichothecenes upregulated the expression of the *gpx4b* gene during the 4 weeks of T-2 toxin exposure. The expression of these genes is regulated through the nuclear factor-erythroid 2-related factor-2 (Nrf2)–antioxidant response element (ARE) pathway (Köhle and Bock 2007; Suzuki and Yamamoto 2015), which responses to emerging oxidative stress (Jennings et al. 2013). DON causes oxidative stress, as was found by Šišperová et al. (2015) in rainbow trout. But, an in vitro study using fish cell lines, including carp brain (CCB) origin lines, found that DON treatment reduced reactive oxygen substances (ROS) production in all cell lines, which is probably due to the activity of antioxidant enzymes. Fish cell lines showed species-related endpoint sensitivities, where rainbow trout appeared to be the most sensitive one (Pietsch et al. 2011). Also, Sanden et al. (2012) showed an elevated level of *cyp1A* mRNA in zebrafish (*Danio rerio*) liver. The effect of FB_1_ on the induction of oxidative stress and antioxidant defence mechanism is known (da Silva et al. 2018), but this effect was not described previously in fish species. OTA promotes lipid peroxidation and oxidative stress (Sava et al. 2006), as was found in zebrafish embryos (Tschirren et al. 2018).

There is a need to investigate the effects of the most relevant mycotoxins in fish species with economic impact, like the common carp. Currently, there is no comprehensive knowledge of DON, or T-2 toxin, FB_1_ and OTA exposure in fish species (Pietsch 2019). The purpose of the present study was to evaluate the short-term effects of a single dose of artificially contaminated feed with DON and its active metabolite 15-acetyl DON (15AcDON), or T-2 toxin and its active metabolite HT-2 toxin, FB_1_ and OTA, in common carp juveniles. Parameters of lipid peroxidation, the antioxidant system and gene expression of Kelch-like ECH-associated protein 1 (*Keap1*), *Nrf2*, and *gpx4a*, and *gpx4b* were followed in 24 h.

## Material and methods

### Experimental design

A total of 132, 1-year-old common carp juveniles (Szarvasi P34 hybrid) was obtained from a commercial fish farm (ÖKO 2000 Ltd., Akasztó, Hungary). After a week of acclimatization period, animals were divided randomly into six treatment groups (control, methyl orange dye control, 0.98 mg T-2 + 0.84 mg HT-2 toxin, 1.68 mg DON + 0.07 mg 15AcDON, 1.96 mg FB_1_,and 1.85 mg OTA kg^−1^ bw, respectively) into six aquaria (150 L each; 22 fishes per aquarium).

Each aquarium was used in a semi-static system with de-chlorinated tap water. For the maximisation of the oxygen level, the water in the aquariums was aerated continuously. The control group received methyl orange dyed (1% w/w) control feed by gavage with the same amount (0.5 g) than the other fishes for the determination of the transit time of feed particles, and the transit time was determined as the period after bolus treatment up to the first occurrence of coloured excreta, which was checked in 30-minute periods. The water temperature was 19 ± 1 °C during the experiment. The light regimen was maintained at a 12:12 h light:dark schedule. The fish bodyweight was 35.92 ± 2.82 g at the start of the trial. The mycotoxin doses were mixed with the same amount of water and ground extruded growth feed for carp (GARANT Aqua Classic™, Garant-Tiernahrung GmbH, Pöchlarn, Austria). The single oral dose of feed was given by gavage for the treatment, which was required to apply the exact amount of feed containing the predicted dose of mycotoxins per kg bodyweight. The predicted dose was 2 mg kg^−1^ bw for each mycotoxin, which is higher than the previously used ones. Based on the average daily feed intake of a carp juveniles (2.5% of metabolic bw) (Csengeri et al. 2013), this dose means approximately 200 mg kg^−1^ feed. These high doses of mycotoxins were selected for the induction of short-term effects of mycotoxins, and nearly the same measured dose was used to compare their effect on the measured parameters. Liver samples were taken from one fish from all groups at the start, six carps of each experimental group, except from T-2 toxin group at 16 h and 24 h, where five samples were taken due to mortality at every 8 h during a 24-h long experimental period into liquid nitrogen and stored at − 70 ^°^C until analysis. No sample was taken from the methyl orange dye fed group during the trial. Nutrient content (on dry matter basis) of the diet was 30% crude protein, 7% crude fat, 5% crude fibre, 7.5% crude ash and 51.5% nitrogen-free extract, respectively. Measured mycotoxin concentrations of the commercial diet were T-2 toxin: < 0.02 mg kg^−1^, HT-2 toxin: < 0.02 mg kg^−1^, DON: < 0.02 mg kg^−1^, 15AcDON: < 0.02 mg kg^−1^, FB_1_: < 0.003 mg kg^−1^ and OTA: < 0.01 mg kg^−1^.

### Production of mycotoxins and analyses

DON was produced by *Fusarium graminearum* (NRRL 5883), and T-2 toxin by *Fusarium sporotrichioides* (NRRL 3299) strains on corn substrate; fumonisin B_1_ was produced by *Fusarium verticillioides* (MRC 826), and ochratoxin A was by *Aspergillus westerdijkiae* (NRRL 3174) according to Fodor et al. (2006, 2008).

DON and 15AcDON content of the feed was determined according to Pussemier et al. (2006), and T-2 and HT-2 toxin concentrations were measured based on the method of Trebstein et al. (2008), while FB_1_ concentration was determined by the method of Trucksess et al. (1995), and OTA according to the method of Visconti et al. (1999) using HPLC method with immunoaffinity clean-up.

### Sampling and biochemical determinations

Animals were over anesthetized with cloves oil, and all individuals were decapitated before sample collection. Liver samples were taken into 1.5-ml collection tubes, frozen in liquid nitrogen, and stored at − 70 °C until analysis.

The amount of conjugated dienes (CD) and trienes (CT), as markers of the initiation phase of lipid peroxidation, was measured according to the AOAC (1984). Malondialdehyde (MDA), a meta-stable end product of lipid peroxidation, concentration was determined by the method of Botsoglou et al. (1994). Reduced glutathione (GSH) concentration was measured as described by Rahman et al. (2007), and the activity of glutathione-peroxidase (GPx) was determined, according to Lawrence and Burk (1976). MDA was measured in the native 1:9 homogenate in isotonic saline (0.65% w/v NaCl), while the other parameters in the 10,000 g supernatant fraction of the homogenates.

GSH content and activity of GPx were calculated to 1-g protein content of the 10,000 g supernatant fraction of liver homogenate, which was measured using Folin-phenol reagent (Lowry et al. 1951).

### RNA isolation, reverse transcription and qPCR

Total RNA extraction was performed with Trizol reagent (Molecular Research Centre, Cincinnati, OH, USA) in Phase Lock Gel tubes (5Prime GmbH, Hamburg, Germany) from 6-mg liver homogenates, according to the phenol-chloroform phase separation method by the instructions of the manufacturer. RNA was DNase treated according to the suppliers’ protocol (Thermo Fisher Scientific, San Jose, CA, USA) to avoid any genomic DNA contamination. The quality and integrity of total RNA were verified by agarose gel electrophoresis and by NanoPhotometer (Implen GmbH, Munich, Germany) measurement. All samples were accepted with the ratios of absorption 260:280 nm higher than 2.0. A standard protocol was used for cDNA production with RevertAID Reverse transcriptase (Thermo Fisher Scientific, San José, CA, USA) and random nanomer primer from 1 μg of total RNA. The primers used for the quantification of the mRNA transcriptional levels of the target and endogenous control genes were chosen based on the literature (Hermesz and Ferencz 2009; Jiang et al. 2015) and are shown in Table 2.

**Table 2:** Primers of target (*gpx4a*, *gpx4b*, *nrf2*, *keap1*) and endogenous control (*β-actin*) genes

Gene	Forward (5′-3′)	Reverse (5′-3′)	Accession No.
*β-actin*	GCAAGAGAGGTATCCTGACC	CCCTCGTAGATGGGCACAGT	XM_019103102.1
*gpx4a*	GGAACCAGGAACAAATTCCC	AGATCCTTCTCCACCACGCTTG	FJ656211.1
*gpx4b*	CTACAAGGCAGAGTTTGACCTC	CTTGGATCGTCCATTGGTCC	FJ656212.1
*nrf2*	TTCCCGCTGGTTTACCTTAC	CGTTTCTTCTGCTTGTCTTT	JX462955
*keap1*	GCTCTTCGGAAACCCCT	GCCCCAAGCCCACTACA	JX470752

The real-time PCR was carried out in pooled samples from equal amounts of cDNA per 6 individuals for each sampling point per treatment. The gene expression measurements were carried out in pooled samples with five technical replicates. Based on our preliminary trials, the actual values did not differ significantly with the individual sample determinations; therefore, it seems useful for gene expression analyses. In the qPCR reactions, Maxima SYBR Green qPCR Master Mix (2×) reaction mixture, (Thermo Fisher Scientific, Budapest, Hungary), 2.4 μM primer in each and 2 μl template cDNA (~ 100 ng) were used for each reaction in a final volume of 12.5 μl. Also, no template controls were performed for each primer pair. The PCR profile for *gpx4a* and *gpx4b* target genes consisted of 95 °C for 10 min, and 95 °C 15 s, 55 °C 30 s and 70 °C 30 s for 45 cycles. For *nrf2* and *keap1* target genes 95 °C for 10 min, and 95 °C 15 s, 60 °C 30 s and 70 °C 30 sec for 45 cycles, where SYBR Green signal was detected at the end of the extension period (Step One Plus™ Real-Time PCR, Thermo Fisher Scientific, Budapest, Hungary). The amplified products were verified by melting curve analysis and gel electrophoresis. The threshold cycle (Ct) of target genes (*gpx4a* and *gpx4b*) and endogenous control gene (*β-actin*) was determined by StepOne™/StepOnePlus™ Software v2.2 (Thermo Fisher Scientific, Budapest, Hungary). The delta Ct values (ΔCt) and relative quantification (RQ = 2ˆ-∆∆Ct) values were calculated by the formula described by Livak and Schmittgen (2001).

### Statistical methods

MedCalc for Windows, version 16.4.1 (MedCalc Software, Ostend, Belgium) was used for the statistical analysis of data. The normality of parameters’ distribution was tested by Kolmogorov-Smirnov test with Lilliefors significance correction, and to confirm for homogeneity of variance, Levene’s test was used. All data meeting both conditions were compared using one-way analysis of variance (ANOVA). The significance of differences between groups was estimated using a post-hoc Student-Newman-Keuls test (*p* < 0.05).

Otherwise, a non-parametric Kruskal-Wallis test with pairwise comparisons was used (*p* < 0.05). Data are presented as mean ± standard deviation (SD).

## Results

Mortality was only found in the T-2 toxin-treated group, where it was 19.04% during the 24 h, while no mortality occurred in the other groups.

Conjugated dienes increased significantly in the case of DON and OTA treatment as compared to control 16 h after exposure (Table 3). Conjugated trienes showed a significant increase as the effect of DON treatment when compared to the control after 16 h (Table 3). Mycotoxin exposure increased the CD value as compared to the initial value in different sampling times up to 16 h. Still, it was higher only in the FB_1_-treated group at 24 h. CT value was higher than the initial value only in the OTA group at 8 h (Table 3).

**Table 3:** Effect of DON, T-2 toxin, OTA or FB_1_ on conjugated dienes and trienes in carp liver (mean ± SD; *n* = 6, except in T-2 group at 16 h and 24 h, where *n* = 5)

Conjugated dienes (OD 232 nm)†				
	0 h	8 h	16 h	24 h
Control	0.310A ± 0.061	0.369AB ± 0.098	0.270^a^A ± 0.025	0.397B ± 0.081
DON		0.472AB ± 0.074	0.615^c^B ± 0.179	0.568AB ± 0.313
T-2		0.532B ± 0.169	0.393^ab^A ± 0.024	0.378A ± 0.065
OTA		0.547B ± 0.101	0.490^bc^B ± 0.148	0.336A ± 0.024
FB_1_		0.462B ± 0.081	0.470^ab^B ± 0.118	0.551B ± 0.121
Conjugated trienes (OD 268 nm)				
Control	0.153A ± 0.032	0.180 ± 0.047	0.130^a^ ± 0.011	0.183 ± 0.042
DON		0.158 ± 0.027	0.216^b^ ± 0.057	0.198 ± 0.110
T-2		0.181 ± 0.056	0.136^a^ ± 0.019	0.133 ± 0.019
OTA		0.186B ± 0.037	0.161^ab^AB ± 0.049	0.124A ± 0.010
FB_1_		0.160 ± 0.034	0.153^ab^ ± 0.039	0.189 ± 0.038

^a, b^Values with different superscripts within columns mean significant difference (*p* < 0.05) between treatment groups

^A, B^Values with a different capital letter within a row mean significant difference between sampling times (*p* < 0.05)

Statistical analysis by one-way ANOVA with a parametric post-hoc Student-Newman-Keuls test was done.

†In the case of this parameter, a non-parametric Kruskal-Wallis test was done

Significantly higher MDA concentration values were measured in the case of FB_1_ treatment as compared to the control after 24 h of exposure (Table 4). Mycotoxin exposure did not change the MDA concentration as compared to the initial value in different sampling times, except in FB_1_-treated group was higher at 24 h (Table 4).

**Table 4:** Effect of DON, T-2 toxin, OTA or FB_1_ on malondialdehyde (MDA) content (μmol/g wet weight) in carp liver (mean ± SD; *n* = 6, except in T-2 group at 16 h and 24 h, where *n* = 5)

Malondialdehyde content (μmol/g wet weight)				
	0 h	8 h	16 h	24
Control	14.72A ± 3.28	14.48 ± 2.81	12.62 ± 3.27	13.69^a^ ± 4.87
DON		14.67 ± 1.76	21.31 ± 5.51	20.95^ab^ ± 8.57
T-2		17.45 ± 4.52	17.64 ± 4.47	15.91^a^ ± 2.07
OTA		18.32 ± 4.28	17.78 ± 4.50	14.47^a^ ± 1.52
FB_1_		14.88A ± 4.62	16.62A ± 3.83	23.47^b^B ± 3.41

^a, b^Values with different superscripts within columns mean significant difference (*p* < 0.05) between treatment groups

^A, B^Values with a different capital letter within a row mean significant difference between sampling times (*p* < 0.05)

Statistical analysis by one-way ANOVA with parametric post-hoc Student-Newman-Keuls test was done

Parameters of the antioxidant defence showed a rapid and marked response to ROS formation in the mycotoxin-treated groups. Significantly higher GSH concentrations were measured in T-2 toxin, and FB_1_-treated groups at 8 h, and as an effect of T-2 toxin at 16 h of mycotoxin exposure as compared to the control (Table 5). T-2 toxin and FB_1_-treated groups showed significantly higher GSH values than the initial value at all sampling times and also in the control group at 24 h (Table 5).

**Table 5:** Effect of DON, T-2 toxin, OTA or FB_1_ on reduced glutathione (GSH) concentration and glutathione peroxidase (GPx) activity in carp liver (mean ± SD; *n* = 6, except in T-2 group at 16 h and 24 h, where *n* = 5)

GSH (mmol/g 10,000 g supernatant protein)				
	0 h	8 h	16 h	24 h
Control	1.40A ± 0.36	1.93^a^AB ± 0.56	1.37^a^A ± 0.39	2.11B ± 0.45
DON		2.09^a^A ± 0.31	3.32^b^B ± 1.17	3.67B ± 1.49
T-2		3.62^b^B ± 0.85	3.24^ab^B ± 1.01	3.18B ± 0.76
OTA		2.32^a^AB ± 0.21	2.94^ab^B ± 0.73	2.84B ± 1.33
FB_1_		3.04^b^B ± 0.84	2.96^ab^B ± 1.68	3.05B ± 0.94
GPx (U/g 10,000 g supernatant protein)				
Control	1.45A ± 0.21	2.08^a^B ± 0.71	1.34^a^A ± 0.47	2.66B ± 0.54
DON		2.90^ab^B ± 0.46	4.15^b^B ± 1.21	3.83B ± 1.78
T-2		5.42^c^C ± 1.44	3.54^ab^B ± 1.18	3.07B ± 1.07
OTA		3.15^**a**b^B ± 0.31	3.78^ab^B ± 0.93	2.81B ± 1.08
FB_1_		4.52^bc^B ± 1.59	4.11^b^B ± 2.45	3.01AB ± 0.92

^a, b^Values with different superscripts within columns mean significant difference (*p* < 0.05) between treatment groups

^A, B^Values with a different capital letter within a row mean significant difference between sampling times (*p* < 0.05) as a result of non-parametric Kruskal-Wallis test

Statistical analysis by one-way ANOVA with parametric post-hoc Student-Newman-Keuls test was done

Similarly to the changes in GSH concentrations, the GPx activities in T-2 toxin and FB_1_-treated groups were significantly higher at 8 h, as compared to the control, and in the case of DON and FB_1_ treatments at 16-h sampling (Table 5). Comparison of GPx activity in different sampling times showed that as compared to the initial value, it was higher in all groups except in control at 16 h (Table 5).

The relative expression of *keap1* gene was significantly lower when compared to the control as an effect of T-2 toxin OTA and FB_1_ treatment at 8 h, and lower values were observed as an effect of DON, OTA and FB_1_ also at 16-h sampling; however, by the 24 h, no significant changes were seen. Comparison of *keap1* gene expression in different sampling times showed that as compared to the initial value, it was lower in all groups at 8 h and 16 h except in control and T-2 toxin-treated and control groups at 24 h (Table 6).

**Table 6:** Effect of DON, T-2 toxin, OTA or FB_1_ on relative gene expression of *keap1* and *nrf2* in carp liver (mean ± SD; *n* = 6, except in T-2 group at 16 h and 24 h, where *n* = 5 in a pool, equal amounts of cDNA)

Gene expression of *keap1*				
	0 h	8 h	16 h	24 h
Control	1.00B ± 0.01	0.68^b^A ± 0.18	0.86^b^AB ± 0.16	0.79^ab^AB ± 0.35
DON		0.45^ab^A ± 0.14	0.51^a^A ± 0.13	0.44^a^A ± 0.10
T-2		0.42^a^A ± 0.09	0.70^ab^A ± 0.15	1.10^b^B ± 0.28
OTA		0.21^a^A ± 0.10	0.49^a^B ± 0.06	0.60^a^B ± 0.14
FB_1_		0.36^a^A ± 0.05	0.61^a^A ± 0.17	0.44^a^A ± 0.12
Gene expression of *nrf2*				
Control	1.00B ± 0.01	0.62^a^A ± 0.11	1.29^c^C ± 0.32	0.42^ab^A ± 0.10
DON		0.92^b^B ± 0.05	0.33^a^A ± 0.07	0.37^a^A ± 0.13
T-2		0.58^a^B ± 0.14	0.32^a^A ± 0.09	0.55^b^B ± 0.07
OTA		0.48^a^B ± 0.16	0.27^a^A ± 0.09	0.41^ab^AB ± 0.12
FB_1_		0.49^a^A ± 0.12	0.61^b^A ± 0.10	0.57^b^A ± 0.12

^a, b^Values with different superscripts within columns mean significant difference (*p* < 0.05) between treatment groups

^A, B^Values with a different capital letter within a row mean significant difference between sampling times (*p* < 0.05)

Statistical analysis by one-way ANOVA with a parametric post-hoc Student-Newman-Keuls test was done

†In the case of this parameter, a non-parametric Kruskal-Wallis test was done

The expression of *nrf2* increased significantly as the effect of the DON at 8-h sampling. Still, it decreased in all mycotoxin-treated groups at 16 h of exposure when compared to the control (Table 6). Comparison of *nrf2* gene expression in different sampling times showed that as compared to the initial value, it was lower in all groups except in the DON group at 8 h and in control at 16 h (Table 6).

The expression of *gpx4a* and *gpx4b* genes showed a dual response during the 24 h. At 8-h sampling, downregulation was observed in the case of both genes in DON, FB_1_, and OTA-treated groups, but an upregulation was found in the case of the T-2 toxin-treated group as compared to the control. At 16 h, upregulation was seen in the DON, T-2 toxin and FB_1_, and at 24 h in the DON and T-2 groups. Expression of *gpx4b* downregulated at 8 h, except in the T-2 toxin-treated group, and upregulation was observed as an effect of T-2 at 24 h (Table 7).

**Table 7:** Effect of DON, T-2 toxin, OTA or FB_1_ on relative gene expression of *gpx4a* and *gpx4b* in carp liver (mean ± SD; *n* = 6, except in T-2 group at 16 h and 24 h, where *n* = 5 in a pool, equal amounts of cDNA)

Gene expression of *gpx4a*				
	0 h	8 h	16 h	24 h
Control	1.00A ± 0.01	4.88^b^C ± 0.73	2.11^a^B ± 0.52	1.19^a^A ± 0.33
DON		1.18^a^A ± 0.48	1.22^a^A ± 0.37	4.76^bc^B ± 1.15
T-2		7.18^c^C ± 1.20	3.40^b^B ± 0.94	13.66^d^D ± 2.26
OTA		0.95^a^A ± 0.32	1.41^a^A ± 0.50	6.30^c^B ± 0.60
FB_1_		1.20^a^A ± 0.47	6.60^c^C ± 0.64	3.07^ab^B ± 0.38
Gene expression of *gpx4b*				
Control	1.00A ± 0.00	3.51^b^C ± 0.92	1.78^ab^B ± 0.41	1.36^a^AB ± 0.29
DON		1.14^a^A ± 0.53	0.83^a^A ± 0.41	2.30^ab^B ± 0.43
T-2		2.29^ab^BC ± 1.18	1.70^ab^AB ± 0.45	± 0.79
OTA		1.19^a^A ± 0.87	1.41^ab^AB ± 0.91	2.31^ab^B 0.53
FB_1_		1.47^a^AB ± 0.30	2.08^b^B ± 0.70	1.61^a^AB ± 0.19

^a, b^Values with different superscripts within columns mean significant difference (*p* < 0.05) between treatment groups

^A, B^Values with a different capital letter within a row mean significant difference between sampling times (*p* < 0.05)

Statistical analysis by one-way ANOVA with a parametric post-hoc Student-Newman-Keuls test was done

## Discussion

The transit time of feed particles in the gut was 16 h in the current study, as was determined by methyl orange dye in the control group. Fish are cold-blooded animals; thus, their metabolism, including gut passage, secretion and activity of digestion enzymes depends on the environment, namely water temperature. In warmer water, their metabolism (Farkas et al. 1980) and mitochondrial activity (Guderley 2003) are higher compared to lower environmental temperature.

A total of 19.04% mortality was observed in the T-2 toxin-treated group in 24-h exposure, which confirms that T-2 toxin is the most toxic trichothecene mycotoxin (Bamburg et al. 1968). Anater et al. (2016) summarised the adverse effects of mycotoxins, including apoptosis in zebrafish (Yuan et al. 2014), which can be concerned with the weak survival of the common carp juveniles.

The amount of CD and CT, markers of the initial phase of lipid peroxidation, elevated in DON- and OTA-treated groups. Elevated CT values were found as an effect of DON at 16 h, but not at 24 h. It means that the initiation phase of lipid peroxidation was induced by DON and OTA during the period of the study, in particular at 16 h when the mycotoxins absorbed from the gastrointestinal tract, according to the transit time of feed particles. The termination phase marker of lipid peroxidation, MDA, was slightly affected by mycotoxin exposure; only FB_1_ caused effect at 24-h sampling. However, a higher tendency of values was observed at 16 h in all mycotoxin-treated groups. These results suggest that significant elevation of ROS occurred in the liver as the effect of the single oral dose of mycotoxins. The initial phase of lipid peroxidation elevated quickly and exceedingly as the effect of DON or OTA, but no such effect was caused by T-2 toxin or FB_1_. This difference among the mycotoxins was probably caused by their different rate of absorption and metabolism. However, the end product of peroxidative processes, MDA, elevated only as an effect of FB_1,_ which means that the ROS production decreased as a function of time, or as an effect of the rapid activation of antioxidant defence.

This supports that the glutathione redox system, as the first line of antioxidant defence, responded quickly and firmly as marked elevations were observed as an effect of T-2 toxin and FB1 at 8 h, in which groups lipid peroxidation did not initiate or reach its termination phase up to the end of this short-term study. GSH content elevated in the T-2 group after 16 h, and at the same time, GPx elevated in DON and FB_1_ groups as an effect of mycotoxin exposure. However, these parameters turned back to the control level after 24 h, which can be concerned with the reliable defence system against ROS forming, which was maintained within the transit time of the feed. This result supports our research group’s previous findings with feeding lower doses of T-2 toxin (0.52 or 2.45 mg kg^−1^ feed) in common carp (Balogh et al. 2009) that resulted in elevated levels of GSH and GPx activity after 7 and 28 days of mycotoxin exposure (Balogh et al. 2009), and also a short-term study (Pelyhe et al. 2016b) with lower levels of T-2 toxin and DON (0.5, 0.33 or 1.82 mg T-2 toxin kg^−1^ bw and 0.13, 0.31 or 1.75 mg DON kg^−1^ bw). However, these changes are similar to another long-term study with common carp juveniles, where the mortality rose; however, the activity of the antioxidant enzymes decreased in 4 weeks (Pelyhe et al. 2016a). There were some changes even in the control group; for instance, GSH content was higher than the initial value up to 24 h. This increase can be explained with the absorption of amino acids from feed up to about 16 h and a higher rate of GSH synthesis when a higher amount of amino acids are available. GPx activity showed different changes in the control group as a function of time, probably due to the availability of the co-substrate GSH.

Gene expression of *gpx4a* and *gpx4b* showed a dual response during the 24 h. A downregulation occurred after 8 h, except as an effect of T-2 toxin. Later, at 16 h, significant elevations were observed as the effect of T-2 toxin and FB_1_, and after 24 h of exposure as an effect of DON and OTA, which means that T-2 toxin and FB_1_ have earlier, and more pronounced effect on the activation of glutathione redox system than DON and OTA. In contrary to the mycotoxin-treated groups, *gpx4a* and *gpx4b* expression showed an early response at 8 h but decreased later. This effect cannot be explained based on the results of the present study; it requires further research but probably has a connection with the different rates of absorption of nutrients from the feed. The more marked lipid peroxidation supports the changes in gene expression profile by these mycotoxins, while the two others activated the antioxidant defence; therefore, no measurable lipid peroxidation activated. ROS formation and oxidative stress caused by the mycotoxins mediate through the redox-sensitive transcription factors which are involved in the activation of the antioxidant system (Jennings et al. 2013). This mechanism is regulated by the Keap1–Nrf2–antioxidant response element (ARE) pathway (Köhle and Bock 2007). However, Keap1 independent pathways are also possible, which were found in mammals (Bryan et al. 2013), but these pathways are not known in fishes yet. As an effect of the applied doses of mycotoxins, downregulation was observed at 8 h and 16 h after the mycotoxin exposure in the case of the expression of the *keap1* gene encoding the Keap1 protein, which is the negative regulator of Nrf2. However, the expression of *nrf2*, encoding the Nrf2 protein, which is responsible for the regulation of several antioxidant genes, was also downregulated at 16 h in all mycotoxin-treated groups. These results suggest that the effect of the applied dose of mycotoxins caused an enhanced formation of reactive oxygen species. Still, these redox changes downregulated the gene expression of *keap1* and *nrf2*, which suggests that activation of antioxidant defence was possibly regulated through Keap1–Nrf2 independent pathways. The findings support that at the same time, the *gpx4* genes overexpressed. However, this result can be explained by the fact that *nrf2* mRNA and Nrf2 protein expression are modified by microRNAs, which may stabilise the *nrf2* mRNA and improve the Nrf2 protein expression even if *nrf2* gene expression is downregulated; therefore, after the Nrf2 translocation to the nucleus, it may induce the gene expression of the antioxidant gene cluster, including *gpx4* (Tonelli et al. 2018).

In conclusion, the short-term effect of T-2 was the most marked as it caused the mortality of some fish, and it is also the most toxic mycotoxin applied in a setup where the doses were the same. T-2 toxin and FB_1_ induced oxidative stress, and DON activated the antioxidant response most effectively. The effect of FB_1_ and OTA was also remarkable but less pronounced at the single oral doses used.

To our knowledge, this is the first publication, which describes the effects of such a wide range of the most common and relevant mycotoxins in common carp, focusing on oxidative stress and the antioxidant system in the liver, which can explain the liver damage as an effect of different mycotoxins. The results showed an early response to the mycotoxins at the high level of contamination, but moderate of low levels of exposure for a long period possibly can cause the same effect as was found in some previous studies. The effect of natural and synthetic antioxidants as useful tools against the oxidative stress caused by mycotoxin exposure is not known in fishes, and it requires further investigations.
